# A Novel α/β Hydrolase Domain Protein Derived From *Haemonchus contortus* Acts at the Parasite-Host Interface

**DOI:** 10.3389/fimmu.2020.01388

**Published:** 2020-06-30

**Authors:** Mingmin Lu, Xiaowei Tian, Ai-Ling Tian, Charles Li, Ruofeng Yan, Lixin Xu, Xiaokai Song, Xiangrui Li

**Affiliations:** ^1^MOE Joint International Research Laboratory of Animal Health and Food Safety, College of Veterinary Medicine, Nanjing Agricultural University, Nanjing, China; ^2^State Key Laboratory of Veterinary Etiological Biology, Key Laboratory of Veterinary Parasitology of Gansu Province, Lanzhou Veterinary Research Institute, Chinese Academy of Agricultural Sciences, Lanzhou, China; ^3^Animal Biosciences and Biotechnology Laboratory, Beltsville Agricultural Research Center, Agricultural Research Service, U.S. Department of Agriculture, Beltsville, MD, United States

**Keywords:** α/β-hydrolase, *H. contortus*, excretory and secretory protein, immunomodulator, parasite-host interaction

## Abstract

The α/β-hydrolase domain (ABHD) proteins belonging to α/β-hydrolase (ABH) superfamily are ubiquitously distributed throughout all the organisms, and their functional roles have been implicated in energy metabolism, cell signaling, growth and development. In our preliminary work, we identified a novel ABHD protein derived from *Haemonchus contortus* excretory-secretory (ES) proteins (HcESPs) that interacted with host T cells. Here, we demonstrated that *H. contortus* ABHD (HcABHD) protein, expressed in all life-cycle stages of *H. contortus*, is a mammalian ABHD17 homolog with immunodiagnostic utility and lipase activity. Given its catalytic activities and immunomodulatory potentials, we further investigated the functional diversity of HcABHD as an individual ES protein in parasite-host interactions. HcABHD protein may serve as depalmitoylase or thioesterase to suppress cell viability, inhibit cell proliferation, induce intrinsic and extrinsic T cell apoptosis, and cause cell cycle arrested at G1 phase. Moreover, recombinant HcABHD stimuli exerted critical controls on T cell cytokine production profiles, predominantly by inhibiting the secretions of interleukin (IL)-4, interferon-gamma (IFN-γ) and transforming growth factor-beta (TGF-β) 1, and promoting IL-10 production. As the immunomodulator acting at the parasite-host interface, HcABHD protein may have potential applications for the vaccine development of therapeutic intervention. Together, these findings may help illuminate the molecular and particularly immunomodulatory aspects of ES proteins and contribute to an enhanced understanding of parasite immune evasion in *H. contortus*-host biology.

## Introduction

The α/β-hydrolase domain (ABHD) proteins which are characterized with beta strands connected by alpha helices in common belong to α/β-hydrolase (ABH) superfamily including esterases, lipases, proteases, peroxidases, dehalogenases, and epoxide hydrolases (1). ABHD proteins are highly conserved and ubiquitously distributed throughout the organisms. To date, at least nineteen mammalian ABHD family members with catalytic properties have been identified, and their physiological functions and biochemical substrates are still being elucidated (2). Human ABHD2 was highly expressed in sperm and functioned as a lipid hydrolase through the activation of steroid hormone progesterone (3). Mouse ABHD4 regulated the metabolism of multiple N-acyl phospholipids in the central nervous system (4). Additionally, human ABHD5, known as Chanarin-Dorfman syndrome protein, acted as a ligand-regulated lipase activator through the interaction with Perilipin to modulate lipolysis (5).

In addition to the engagement in lipid metabolism and metabolic diseases, ABHD enzymes have also been implicated in a wide range of cellular functions including cell migration, proliferation, and cell survival. For instance, stable expression of ABHD2 in LNCaP cells promoted cell proliferation and enhanced cell migration, whilst inhibition of ABHD2 transcription suppressed prostate cancer growth and induced cell apoptosis (6). Besides, ABHD15 is a directly target gene of peroxisome proliferator-activated receptor gamma and blocking ABHD15 expression resulted in apoptosis of 3T3-L1 cells (7). Furthermore, recent studies revealed that ABHD5 regulated autophagy of human colon epithelial cells *via* the interaction with BECN1 (8), whereas ABHD5 expression in colorectal cancer (CRC)-associated macrophages significantly enhanced cell viability, cell cycle, and clone formation of CRC cells (9).

Apart from the broad distribution in mammals, ABHD proteins and its homologs have been sparsely reported in plants and yeasts maintaining lipid homeostasis at the interface of cellular metabolism and signal transduction, as exemplified by *Arabidopsis thaliana* ABHD11 and ABHD5 (10, 11), and *Saccharomyces cerevisiae* ABHD5 homologs (12). Similarly, comparable expressions of ABHD proteins/homologs were also demonstrated in free-living and parasitic parasites such as *Caenorhabditis elegans* ABHD5 (13), *Cryptosporidium parvum* Type II thioesterase (CpTEII) (14) and *Schistosoma japonicum* lysophospholipase (15). Moreover, ABHD proteins were enriched in the excretory and secretory (ES) products or somatic proteome of parasitic nematodes, namely, *Haemonchus contortus* (16), *Heligmosomoides polygyrus* (17), and *Mesocestoides corti* (18). Like the proteases and hydrolase that engage in energy metabolism and signaling, ABHD proteins are postulated to play pivotal roles in parasite development, survival and reproduction *via* the digestion or degradation of endogenous and host lipids (17, 19).

In our previous study, we identified 114 *H. contortus* excretory-secretory (ES) proteins (HcESPs) that interacted with goat T cells by liquid chromatography-tandem mass spectrometry (LC-MS/MS) analysis and *H. contortus* ABHD (HcABHD) protein was ascertained among these interacting proteins (20). Simultaneously, HcESPs stimuli notably induced Fas-engaged intrinsic and extrinsic apoptosis, suppressed T cell proliferation and caused cell cycle arrested *via* limiting Akt/PKB signaling (20). HcESPs contained a variety of modulatory molecules such as kinases, hydrolases, phosphatases, proteases and lipases, whereas the pleiotropic effects of HcESPs were generated by a cascade of individual ES components. Importantly, the exact molecule(s) which regulate with T cell directly/indirectly at the parasite-host interface warrant further investigation. Given the functional diversity of ABHD proteins, particularly its involvement in cell proliferation and apoptosis, HcABHD could be one of these dominated proteins which exerted critical controls on cell death and survival of host key effector cells. Thus, in this study, we aimed to characterize the functional properties of HcABHD protein and elucidate its immunomodulatory trait in *H. contortus*-host interactions.

## Materials and Methods

### Ethics Statement

All experimental protocols were reviewed and approved by the Science and Technology Agency of Jiangsu Province (SYXK (SU) 2010–0005). All animal experiments were performed in strict compliance with the Guidelines of the Animal Welfare Council of China. All efforts were made to minimize the suffering of animals, and daily health checks were performed throughout the experiments.

### Parasite, Animal, and Cell

*H. contortus* strain was maintained and propagated by serial passages in nematode-free goats in the laboratory of Veterinary Parasitology, Nanjing Agricultural University, Nanjing, China. The collection of eggs, L3, xL3, male and female adults of *H. contortus* was performed as previously described (21, 22).

Sprague Dawley (SD) rats (female, ~6 weeks, body weight ~150 g) were purchased from Experimental Animal Center of Jiangsu, Nanjing, China (SCXK 2008-0004). They were raised in a sterilized room with access to sterilized food and water *ad libitum*.

Local crossbred and healthy goats (female, 5–6 months of age) were reared in individually ventilated cages in case to accidental infection with nematodes. They were fed with hay and whole shelled corn, as well as water *ad libitum* in pens. Peripheral venous blood samples (40 mL for each) were obtained by venipuncture from these goats and the isolation of goat peripheral blood mononuclear cells (PBMCs) were managed as previously described (23). Total T cells were sorted from goat PBMCs by the magnetic-activated cell sorting system (MACS, Miltenyi Biotech Inc, Auburn, CA) as described elsewhere (24). Briefly, PBMCs were resuspended to the density of 1 × 10^6^ cells / mL in phosphate buffer saline (PBS) containing 2 mM EDTA and 0.5 % bovine serum albumin (BSA, Sigma-Aldrich, St. Louis, MO, USA). Then every 1 × 10^6^ PBMCs in 100 μL of staining buffer were incubated with 10 μL of mouse anti-bovine CD2 primary antibody (Bio-Rad, Kidlington, UK) which cross-react with goat CD2 T cells at room temperature for 30 min. After two washes in PBS, 1 × 10^7^ total cells in 100 μL of staining buffer were labeled with 10 μL of anti-FITC MicroBeads (Miltenyi Biotech) at room temperature for 15 min. Subsequently, the cell suspensions were loaded on the MACS MS Column (Miltenyi Biotech) placed in the magnetic field of the MACS Separator (Miltenyi Biotech), and magnetically labeled T cells were retained in the column. After removing the column from the MACS Separator, T cells were eluted as the positively selected cell fractions. T cells were then resuspended to a density of 1 × 10^6^ cells / mL in RPMI 1640 (Gibco, Grand Island, NY, USA) containing 100 U/mL penicillin, 100 mg/mL streptomycin (Gibco) and 10% heat-inactivated fetal calf serum (FCS, Gibco) and activated with concanavalin A (ConA, 5 μg/mL) for functional studies. The viability of T cells was >95% as assessed by the trypan blue exclusion test. The purity of isolated T cells was above 95% as measured by flow cytometry (Supplementary Figure 1). Three biological replicates (three goats) were used in each experiment.

### Cloning and Sequence Analysis of HcABHD

Total RNA was isolated from adult *H. contortus* using Trizol reagent (Invitrogen, San Diego, CA, USA) according to the manufacturer's instructions. First-strand cDNA was synthesized from total RNA by reverse transcription polymerase chain reaction (RT-PCR). The DNA fragment encoding HcABHD was amplified with forward primer (5′-3′: CACgaattcATGTCTGGAGTGCAGTGTACTG) and reverse primer (5′-3′: AACctcgagTACGCGGACGCCAATG). The amplified HcABHD gene was cloned into pET28a (+) vector (Invitrogen) and validated by sequence analysis using BLAST. The phylogenetic tree was generated by the Neighbor-Joining method using MEGA X software (25). Prior to the phylogenetic inference, all positions with <80% site coverage were excluded with partial deletion option. The evolutionary distances were extrapolated using the Dayhoff matrix based method (26).

### Expression and Purification of Recombinant HcABHD (rHcABHD) Protein

The rHcABHD were expressed and purified as previously described (27). Briefly, *E. coli* BL21 (DE3) cells containing the reconstructed pET28a-HcABHD plasmid were cultured in Luria-Bertini medium with kanamycin (100 μg/mL, Sigma-Aldrich) at 37°C, and then induced with Isopropyl-β-D-thiogalactopyranoside (1 mM, Sigma-Aldrich) at 37°C for another 5 h to express the rHcABHD protein. The histidine-tagged fusion protein was purified from the supernatant of bacterial lysates using HisTrap HP purification columns (GE Healthcare, Piscataway, NJ, USA). rHcABHD protein was resolved on 12% sodium dodecyl sulfate-polyacrylamide gel electrophoresis (SDS-PAGE) gels for size and purity validation, and the concentration was determined by the Bradford method. Lipopolysaccharide (LPS) was depleted from the rHcABHD protein using Detoxi-Gel Affinity Pak prepacked columns (Thermo Fisher, Rockford, IL, USA). The purified proteins were stored at −80°C until further analysis.

### Generation of Polyclonal Antibody

To generate antigen-specific polyclonal antibodies, 300 μg of rHcABHD protein, blended with Freund's complete adjuvant, was administrated subcutaneously into SD rats for primary immunization. SD rats were later boosted four times with the same dose of rHcABHD emulsified in Freund's incomplete adjuvant at 2-week intervals. One week after last boost, rat sera containing specific anti-rHcABHD antibodies were collected and stored at −80°C for later use.

The sera harvested from goats experimentally infected with *H. contortus* (anti-*H. contortus* serum) were stored at the Veterinary Parasitology Teaching and Research Center of Nanjing Agricultural University, Nanjing, China.

### Immuno-Blot Analysis

rHcABHD and HcESPs proteins were resolved on 12% SDS-PAGE gels, respectively, and electro-transferred onto nitrocellulose membranes. The membranes were blocked with 5% skim (non-fat) milk in TRIS-buffered saline containing 0.1% Tween-20 (TBST) at room temperature for 1 h. The blots with rHcABHD samples were probed with goat anti-*H. contortus* serum (1:500 in TBST) or normal goat serum (control) overnight at 4°C, whereas the blots with HcESPs samples were probed with rat anti-rHcABHD IgG or normal rat IgG (control) overnight at 4°C. After five washes in TBST, the membranes were incubated with horseradish peroxidase (HRP)-conjugated rabbit anti-goat or anti-rat IgG (H+L) secondary antibody (Sigma-Aldrich) in TBST (1:5000) for 1 h at 37°C. Immunoreactions were detected using 3,3′-diaminobenzidine (Sigma-Aldrich) as a chromogenic substrate for 3–5 min color development.

### Transcription of HcABHD Gene in *H. contortus* Life-Cycle Stages

To detect transcriptional expression of HcABHD in *H. contortus* life-cycle stages, total RNA of eggs, L3, xL3, female and male adults were extracted and the resulting cDNAs were synthesized in accordance with the manufacturer's specifications. Transcription analysis of candidate gene was conducted by real-time PCR using QuantStudio 3 System (Applied Biosystems, Carlsbad, CA, USA) with a standard procedure, using specific primers for endogenous reference gene β-tubulin (28) and target gene HcABHD (Supplementary Table 1). The amplification efficiencies and correlation coefficients were verified to be stable and similar, and the relative transcription levels of HcABHD were calculated by the 2^−ΔΔCt^ method. Each experiment was run in triplicate.

### Immunohistochemistry Assays for HcABHD Localization

Freshly collected female and male adults (20 ~ 30 for each) were washed, dehydrated, fixed, embedded and cut into cryostat sections as previous described (22). To block non-specific binding, the cryosections were treated with 10% normal goat serum in PBS containing 0.1% Tween-20 (PBST) at room temperature for 1 h. The sections were then incubated with rat anti-rHcABHD IgG (1:200) or normal rat IgG (control) at 4°C overnight. After three washes in PBST, the sections were then incubated with Cy3-labeled goat anti-rat IgG (1:500) for 1 h at 37°C. Following three washes in PBST, 2-(4-Amidinophenyl)-6-indolecarbamidine dihydrochloride (DAPI, Sigma-Aldrich) were used for DNA staining at room temperature for 5 min. The samples were then immersed into anti-fade medium (Sigma-Aldrich) to prevent fluorescence fading for microscopic examination. Finally, the sections were imaged using LSM710 fluorescence microscope (Zeiss, Jena, Germany) at 60× magnification and digital images were analyzed using ZEN 2012 software (Zeiss).

### Lipase Activity Assay

The lipase activity of rHcABHD was determined using the lipase activity assay kit (Sigma-Aldrich) according to the manufacturer's protocol. For the samples and standards, various concentration of rHcABHD proteins (0, 10, 20, 40, and 80 μg/mL) or standards (0, 2, 4, 6, 8, and 10 nmole/well) were added together with lipase assay buffer into the 96-well plate to bring the volume to 50 μL. For the positive control, 5 μL of the lipase positive control solution was assayed to the wells and adjusted to 50 μL with the lipase assay buffer. Subsequently, 93 μL of lipase assay buffer, 2 μL of peroxidase substrate, 2 μL of enzyme mix and 3 μL of lipase substrate were added to each well to generate 100 μL of reaction mix. The plate was then incubated at 37°C for 20 min and the absorbance (A570) values were read every 5 min. The lipase activity was calculated based on colorimetric detection of various concentrations of glycerol standards. Each experiment was run in duplicate.

### The Interaction of HcABHD Protein With T Cells *in vitro*

The interaction of HcABHD with goat T cells was investigated as previously described (27). Briefly, freshly sorted T cells were incubated in the presence or absence of 10 μg/mL of rHcABHD proteins at 37°C for 2 h. After three washes, T cells were fixed with 4% paraformaldehyde for 20 min at room temperature, permeabilized by 0.5% Triton X-100 in PBST for 5 min and blocked with 4% BSA in PBST for 30 min to minimize background staining. Subsequently, cells were treated with rat anti-HcABHD IgG (1:100) or normal rat IgG (control) in a humidified atmosphere at 37°C for 1 h, followed by staining with Cy3-coupled secondary antibody (1:500). After three washes in PBST, T cells were labeled in Gold Anti-fade mounting solution containing DAPI (Life Technologies, Eugene, OR, USA) for nuclear staining. Immunofluorescence-labeled cells were imaged using LSM780 laser scanning confocal microscope (Zeiss, Jena, Germany) at 100× magnification and digital images were analyzed by Zen 2012 software (Zeiss).

### Cell Viability

The impacts of rHcABHD on goat T cell viability were determined by using the cell counting kit-8 assay (CCK-8; Dojindo, Kumamoto, Japan) as previously described (29). T cells activated with ConA (5 μg/mL) were treated with different concentrations of rHcABHD proteins (0, 10, 20, 40 and 80 μg/mL) at 37°C with 5% CO2 in a humidified atmosphere. Following 24 h-incubation, 10 μL of CCK-8 reagent was incorporated and incubated in the dark for 4 h. The optical density at 450 nm (OD450) was measured using a microplate reader (Bio-Rad, Hercules, California, USA). Three individual experiments, each consisting of three replicates, were carried out.

### Cell Apoptosis Assay

Flow cytometry analysis of T cell apoptosis was performed using the Annexin V-PE kit (BD Biosciences, San Jose, California, USA) as previously described (27). Briefly, T cells were incubated with various concentrations of rHcABHD proteins (0, 10, 20, 40, and 80 μg/mL) followed by staining with annexin V and 7-Aminoactinomycin D (7-AAD) based on the manufacturer's protocol. The PBS-treated goat T cells were served as negative controls. Three independent experiments were performed and each experiment was run in triplicate.

### Cell Proliferation Assay

The detection of cell proliferation was performed by measuring DNA synthesis directly using the Alexa Fluor 647 click-iT plus EdU flow cytometry kit (Thermo Fisher) according to the manufacturer's instruction. At the end of the 12 h co-incubation period, 5-ethynyl-2'-deoxyuridine (EdU, 10 μM) was added to the culture medium for another 12 h incubation in a humidified atmosphere at 37°C with 5% CO2. Subsequently, T cells were harvested, fixed with 4% paraformaldehyde in PBS and permeabilized using Click-iT saponin-based reagent, followed by Click-iT reaction to couple EdU with Alexa Fluor 647 dye. After being washed with 3 mL of 1% BSA in PBS, T cells were treated with 7-AAD staining solution (BD Biosciences). Flow cytometry was used for determination of the percentage of EdU^+^ cells in the population. Three independent experiments were performed and each experiment was run in triplicate.

### Cell Cycle Analysis

Flow cytometry analysis of cell cycle was performed according to manufacturer's DNA staining protocol. During the incubation with rHcABHD stimuli (20 μg/mL) for 24 h, T cells were collected, washed and fixed with ice-cold 75% ethanol every 6 h. After frozen at −20°C for 12 h, cells were then washed twice in PBS to remove the ethanol and resuspended in PI/RNase staining buffer for flow cytometry analysis. Each experiment consisting of three replicates was run in triplicate.

### Transcription Analysis

T cells treated with various concentration of rHcABHD (0, 10, 20, 40, and 80 μg/mL) for 12 h were harvested for the transcription analysis of cell apoptosis, and T cells treated with 20 μg/mL for different times (0, 6, 12, 18, and 24 h) were collected for the transcription analysis of cell cycle. Cells were harvested for total RNA extraction, and cDNA was obtained by RT-PCR. Relative quantification of candidate gene expressions was conducted using previously published primers of endogenous reference genes and target genes (30–34) (Supplementary Table 2). The relative mRNA expression levels of candidate genes were calculated by the 2-^ΔΔCt^ method. Each experiment was run in triplicate.

### Detection of Cytokine Secretions

To determine cytokine secretion levels, fresh isolated T cells activated by ConA (5 μg/mL) were incubated in the presence or absence of rHcABHD (0, 10, 20, 40 and 80 μg/mL) for 24 h in a humidified atmosphere with 5% CO2 at 37°C. The supernatants were collected and assayed for cytokine secretion detection using goat enzyme linked immunosorbent assay (ELISA) kits (Mlbio, Shanghai, China) according to the manufacturer's specifications. The limits of detection were between 2 and 800 pg/mL depending on the analytic assay. Each experiment was run in triplicate.

### Statistical Analysis

One-way analysis of variance (ANOVA) with Dunnett's multiple comparison test was performed for statistical analysis using GraphPad Premier 6.0 software (GraphPad Prism, San Diego, CA, USA). *P* < 0.05 was considered to be statistically significant among group differences. Data were presented as minimum to maximum or mean ± standard deviation (SD).

## Results

### Phylogenetic Analysis and Protein Expression

**Figure 1:**
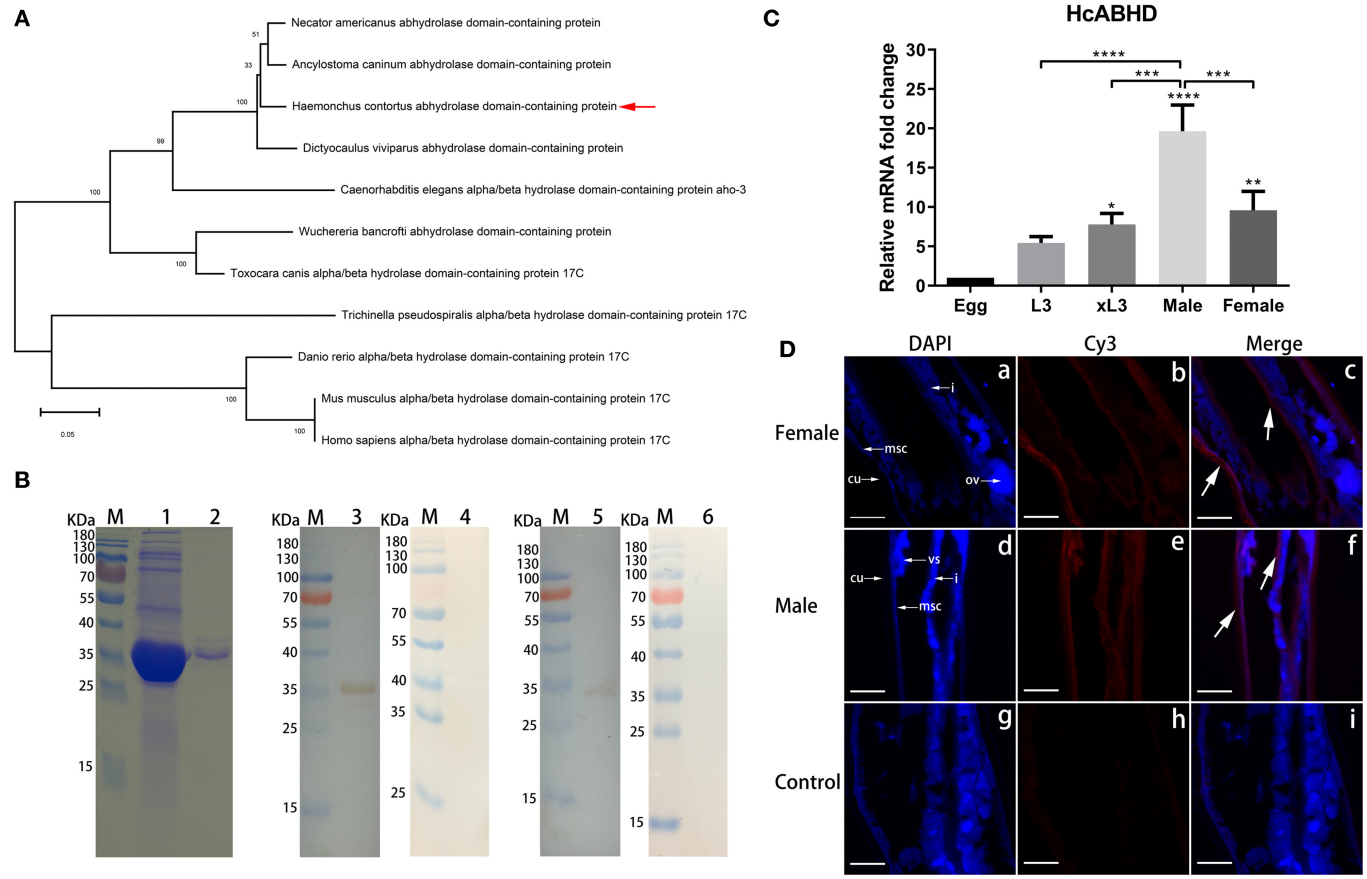
Molecular characterization of HcABHD that was derived from HcESPs**. (A)** Phylogenetic analysis of HcABHD with vertebrate orthologs and parasite homologs. Evolutionary relationships of taxa was inferred using the Neighbor-Joining method with protein sequences including *Haemonchus contortus* (CDJ88804.1), *N. americanus* (XP_013304149.1), *A. caninum* (RCN37647.1), *D. viviparus* (KJH43748.1), *C. elegans* (NP_492210.2), *Wuchereria bancrofti* (EJW78952.1), *Toxocara canis* (KHN80403.1), *Trichinella pseudospiralis* (KRX88540.1), *D. rerio* (NP_956451.1), *M. musculus* (NP_598483.2), and *H. sapiens* (NP_067037.1). Bootstrap support values are shown for each node. The scale bar indicates the number of substitutions per site. **(B)** Acquisition of rHcABHD proteins and Western blot analysis. Lane M: standard protein molecular marker; Lane 1: rHcABHD expressed in the supernatant of cell lysates; Lane 2: SDS-PAGE analysis of purified rHcABHD protein; Lane 3: Immunoblot analysis of rHcABHD using anti-*H. contortus* serum as primary antibody; Lane 4: Immunoblot analysis of rHcABHD using normal goat serum (control) as primary antibody; Lane 5: Immunoblot analysis of HcESPs using rat anti-rHcABHD IgG as primary antibody; Lane 6: Immunoblot analysis of HcESPs using normal rat IgG (control) as primary antibody. **(C)** HcABHD expression in life-cycle stages of *H. contortus*. The transcription levels of HcABHD were determined in eggs, L3, xL3, male and female adults. Data were designated as mean ± SD, *P < 0.05, **P < 0.01, ****P < 0.0001 vs. control group (eggs) and a capped line designated that two groups differed significantly (***P < 0.001, ****P < 0.0001). **(D)** Immunolocalization of native HcABHD protein in male and female adult worms. The immunohistochemistry assays were performed using rat anti-rHcABHD IgG or normal rat IgG (control) as primary antibody. Using anti-rHcABHD IgG as a probe, HcABHD was shown to be highly expressed in the platymyrian muscle cells (msc) under the cuticle (cu) and the intestine (i) of both female (a-c) and male worms (d-f). No labeling was observed in worm sections probed with normal rat IgG (g-i). Ovaries (ov) and seminal vesicle (vs) were also indicated. DAPI (blue) and Cy3-conjugated secondary antibodies (red) were utilized for double staining. Scale bars, 100 μm.

The complete coding sequence of the HcABHD gene (939 bp) was cloned from adult worms, encoding a 313-amino acid protein with predicted molecular mass approximately 34 kDa. We conducted an evolutionary analysis of HcABHD using the Neighbor-Joining method involving 11 amino acid sequences with a total of 270 positions in the final dataset (Figure 1A). Phylogenetic analysis clearly showed an evolutionary relationship of HcABHD with other ABHD orthologs, revealing that HcABHD was closely related to parasitic nematode (*Necator americanus, Ancylostoma caninum* and *Dictyocaulus viviparus*) homologs, but divergent from vertebrate (*Danio rerio, Mus musculusa*, and *Homo sapiens*) orthologs (Figure 1A).

The rHcABHD protein was successfully expressed as histidine-tagged fusion protein in the supernatant of cell lysates. After purification, rHcABHD was visualized by Coomassie Blue staining as a single band on SDS-PAGE gels with a molecular weight of ~36 kDa (Figure 1B, Lane 2). The specificity of the rHcABHD protein was determined by Western blot, probed with anti-*H. contortus* serum or normal goat serum. A single band of ~36 kDa was observed through the specific recognition of rHc-ABHD protein by anti-*H. contortus* serum (Figure 1B, Lane 3), whilst no band was recognized by normal goat serum (Figure 1B, Lane 4). Meanwhile, the native HcABHD protein derived from HcESPs was identified by rat anti-rHcABHD IgG as a single ~34 kDa band (Figure 1B, Lane 5), whereas no positive bands were observed in the control groups (Figure 1B, Lane 6).

### The mRNA Expression of HcABHD in *H. Contortus* Life-Cycle Stages and Immunolocalization

Real-time PCR analysis revealed that mRNA transcripts of HcABHD were detectable in all the tested life-cycle stages of *H. contortus*. The increased expression levels of HcABHD were observed in xL3 (*P* < 0.05), female (*P* < 0.01) and male adults (*P* < 0.0001) compared to that of eggs (Figure 1C). Concurrently, the highest level of HcABHD transcription was revealed in male adults compared to that in xL3 (*P* < 0.001) and female adults (*P* < 0.001) (Figure 1C).

Given that the high transcription levels of HcABHD were detected in adult worms, we performed immunohistochemistry assays to investigate the localization of native HcABHD proteins within *H. contortus* by checking the cryosections of middle region of adult worms. Specific red fluorescence resulting from tagging native HcABHD proteins by rat anti-rHcABHD IgG was observed from the platymyrian muscle cells under the cuticle, as well as the gut region, in both female (Figure 1D, c) and male (Figure 1D, f) adults. However, no red fluorescence was detected in the control sections treated with normal rat IgG (Figure 1D, i).

### Lipase Activity

**Figure 2:**
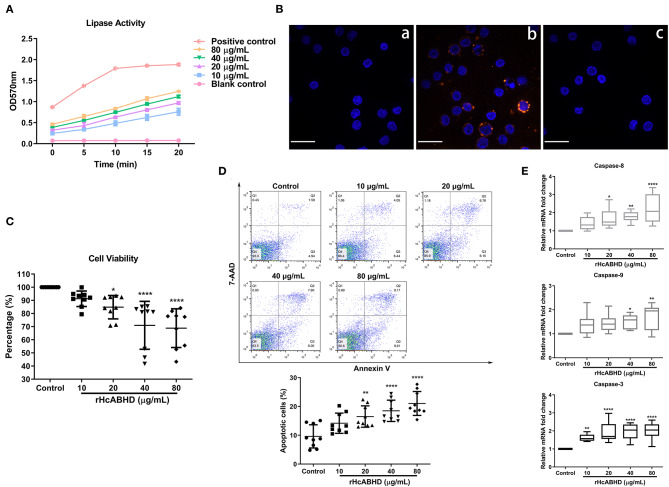
rHcABHD proteins with lipase activity suppressed cell viability and induced apoptosis via the interactions with goat T cells. **(A)** Determination of the lipase activity of rHcABHD. Lipase activity is reported as nmole/min/mL = milliunit/mL. One unit of lipase is the amount of enzyme that generates 1.0 mole of glycerol from triglycerides per minute at 37°C. Data were designated as mean ± SD. Each experiment was run in duplicate. **(B)** Confirmation of the interaction of HcABHD protein with goat T cells. T cells treated in the absence (a) or presence (b) of rHcABHD protein were incubated with rat anti-rHcABHD IgG as primary antibody. T cell pretreated with rHcABHD were incubated with normal rat IgG as primary antibody (c). DAPI (blue) and Cy3-conjugated secondary antibodies (red) were utilized for double staining. Scale bars, 50 μm. **(C)** rHcABHD significantly inhibited T cell viability. Cell viability tests were determined by CCK-8 incorporation, and the cell viability index was determined by calculating the OD450 values of control group (T cells treated with 0 μg/mL rHcABHD) as 100%. Data were designated as mean ± SD, *P < 0.05, ****P < 0.0001 vs. control group. **(D)** Flow cytometry analysis of T cell apoptosis in responses to rHcABHD stimuli. The apoptosis rate was calculated by the percentage of early (AnnexinV^+^7AAD^−^) and late (AnnexinV^+^7AAD^+^) apoptotic T cells. Data are presented as the mean ± SD, **P < 0.01, ****P < 0.0001 vs. control group (T cells treated with 0 μg/mL rHcABHD). **(E)** The mRNA transcripts of caspase-3,−8 and−9 in rHcABHD-stimulated T cells. Data are presented as Minimum to Maximum, *P < 0.05, **P < 0.01, ****P < 0.0001 vs. control group (T cells treated with 0 μg/mL rHcABHD). Results presented here are representative of three independent experiments.

Lipase activity of rHcABHD was determined using a coupled enzyme reaction, which resulted in a colorimetric (570 nm) product proportional to the enzymatic activity present. The results revealed that all the tested concentrations of rHcABHD proteins (10, 20, 40, and 80 μg/mL) had the *in vitro* lipase activities (12.9, 16.6, 18.8, and 20.2 milliunits/mL, respectively) in a dose-dependent manner as compared with the blank control, but with lower enzyme activities than the positive control (52.8 milliunits/mL) (Figure 2A).

#### Binding of rHcABHD Protein to Goat T Cells

Based on our preliminary LC-MS/MS analysis, we next performed immunofluorescent staining assays to verify the *in vitro* interaction of HcABHD protein with goat T cells. Immunocytochemistry assays showed that intense red Cy3-fluorescence resulting from tagging rHcABHD was observed in rHcABHD-treated T cells (Figure 2B, b). Additionally, no red fluorescence was detected in both blank and negative control groups (Figure 2B, a and c). The results presented here further validated the positive interactions between HcABHD protein and host T cells.

### rHcABHD Affected Cell Viability and Induced Cell Apoptosis

Given the modulatory potential of ABHD proteins on cellular development and survival, we next investigated the influence of rHcABHD proteins on T cell viability. The results showed that the viability of goat T cells were dramatically inhibited by the treatments of 20 μg/mL (*P* < 0.05), 40 μg/mL (*P* < 0.0001) and 80 μg/mL (*P* < 0.0001) of rHcABHD proteins (Figure 2C). Based on this finding, an Annexin V-PE/7-AAD double staining kit was employed to evaluate the pro-apoptotic potential of rHcABHD proteins. Flow cytometry results revealed that rHcABHD protein, at the tested concentrations of 20 μg/mL (*P* < 0.01), 40 μg/mL (*P* < 0.0001) and 80 μg/mL (*P* < 0.0001), significantly induced T cell apoptosis *in vitro* as compared with the control group (Figure 2D). Furthermore, transcription analysis of key genes engaged in apoptosis signaling pathways further validated the pro-apoptotic effects of rHcABHD proteins on host T cells. The treatments with 20 μg/mL (*P* < 0.05), 40 μg/mL (*P* < 0.01) and 80 μg/mL (*P* < 0.0001) of rHcABHD significantly upregulated the mRNA transcripts of caspase-8. Simultaneously, 40 μg/mL (*P* < 0.05) and 80 μg/mL (*P* < 0.01) of rHcABHD proteins significantly promoted caspase-9 transcription. Importantly, all the tested concentration of rHcABHD (10, 20, 40 and 80 μg/mL) dominantly enhanced caspase-3 transcription (*P* < 0.01, *P* < 0.0001, *P* < 0.0001 and *P* < 0.0001, respectively) (Figure 2E).

### rHcABHD Protein Inhibited Cell Proliferation and Induced Cell Cycle Arrest

**Figure 3:**
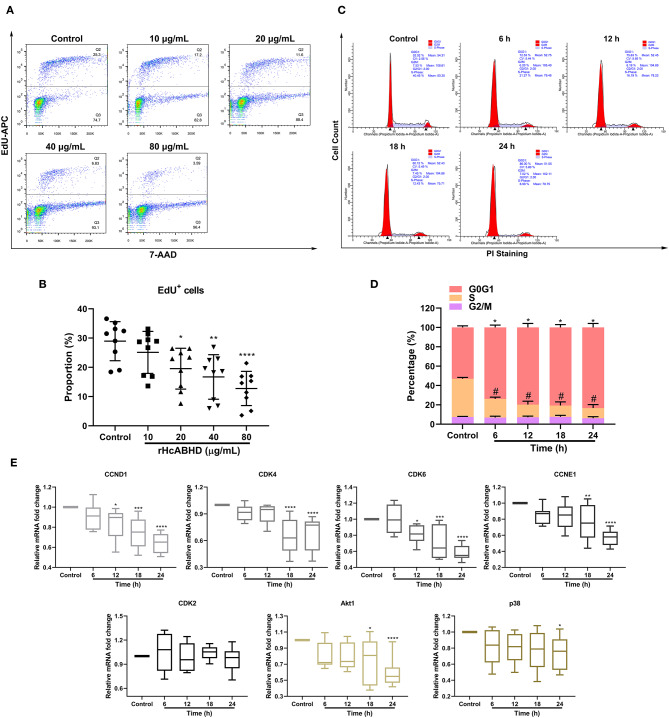
rHcABHD protein suppressed T cell proliferation and induced T cell cycle arrest through Akt/MAPK signaling. **(A)** Determination of T cell proliferation in responses to rHcABHD stimuli. Flow cytometry analysis were performed using 7-AAD and EdU-APC double staining. T cells treated with 0 μg/mL rHcABHD served as control group. **(B)** Statistic analysis of T cell proliferation rate. The proliferation rate was calculated by the percentage of EdU^+^ T cells. Data are presented as the mean ± SD, *P < 0.05, **P < 0.01, ****P < 0.0001 vs. control group (T cells treated with 0 μg/mL rHcABHD). Results presented here are representative of three independent experiments. **(C)** T cell cycle analysis in responses to rHcABHD stimuli. Cell cycle determination of T cells was performed using PI/RNase staining regent by flow cytometry. T cells treated with 20 μg/mL of rHcABHD at 0 h served as control group. **(D)** Statistic analysis of T cell cycle. T cells incubated with rHcABHD stimuli were collected every 6 h. The results demonstrated that rHcABHD caused cell cycle arrest at G0/G1 phase. Results are denoted as mean ± SD, whereas the * (asterisk) for G1 phase determination (*P < 0.05, **P < 0.01) and ^#^ (hashtag) for S phase determination (*P* < 0.05) indicated statistically significant difference to control group (T cells treated with 20 μg/mL of rHcABHD at 0 h). Results presented here are representative of three independent experiments. **(E)** Relative gene transcription levels of CCND1, CCNE1, CDK4/6, CDK2, Akt1, and p38 in rHcABHD-treated T cells. Data are presented as Minimum to Maximum, *P < 0.05, **P < 0.01, ***P < 0.001, ****P < 0.0001 vs. control group (T cells treated with 20 μg/mL of rHcABHD at 0 h). Results presented here are representative of three independent experiments.

As apoptosis, proliferation and cell cycle were interconnected cellular movements, we next evaluated the modulatory potential of rHcABHD stimuli on T cell proliferation and cell cycle. The treatments with rHcABHD proteins (20, 40, and 80 μg/mL) significantly inhibited the proliferation of goat T cells *in vitro* (Figure 3A), as indicated by the decreased percentages of EdU^+^ cells compared with control cells (*P* < 0.05, *P* < 0.01, and *P* < 0.0001, respectively) (Figure 3B). Given that the treatment with 20 μg/mL of rHcABHD had significant biological effects on cell viability, apoptosis and proliferation, we next treated T cells with 20 μg/mL of rHcABHD for cell cycle determination. Here, flow cytometry analysis with PI staining revealed that rHcABHD stimuli induced cell cycle arrest in a time-dependent manner (Figure 3C), as exemplified by the increased percentages of cells in G1 phase at 6, 12, 18 and 24 h (*P* < 0.0001 for all) and the decreased percentages of cells in S phase at 6, 12, 18 and 24 h (*P* < 0.0001 for all) (Figure 3D). Consistent with these findings, transcriptional analysis of key genes in G1/S checkpoints showed that mRNA expression of CCND1 at 12 h (*P* < 0.05), 18 h (*P* < 0.001) and 24 h (*P* < 0.0001), CCNE1 at 18 h (*P* < 0.01) and 24 h (*P* < 0.0001), CDK4 at 18 h (*P* < 0.0001) and 24 h (*P* < 0.0001), and CDK6 at 12 h (*P* < 0.05), 18h (*P* < 0.001), and 24 h (*P* < 0.0001) were significantly downregulated by rHcABHD stimuli, while no significant transcriptional changes of CDK2 were observed at all timepoints (Figure 3E). Furthermore, Akt1 transcription at 18h (*P* < 0.05) and 24 h (*P* < 0.0001), and p38 transcription at 24 h (*P* < 0.05) was notably inhibited by rHcABHD stimuli (Figure 3E).

### Determination of Cytokine Secretions

**Figure 4:**
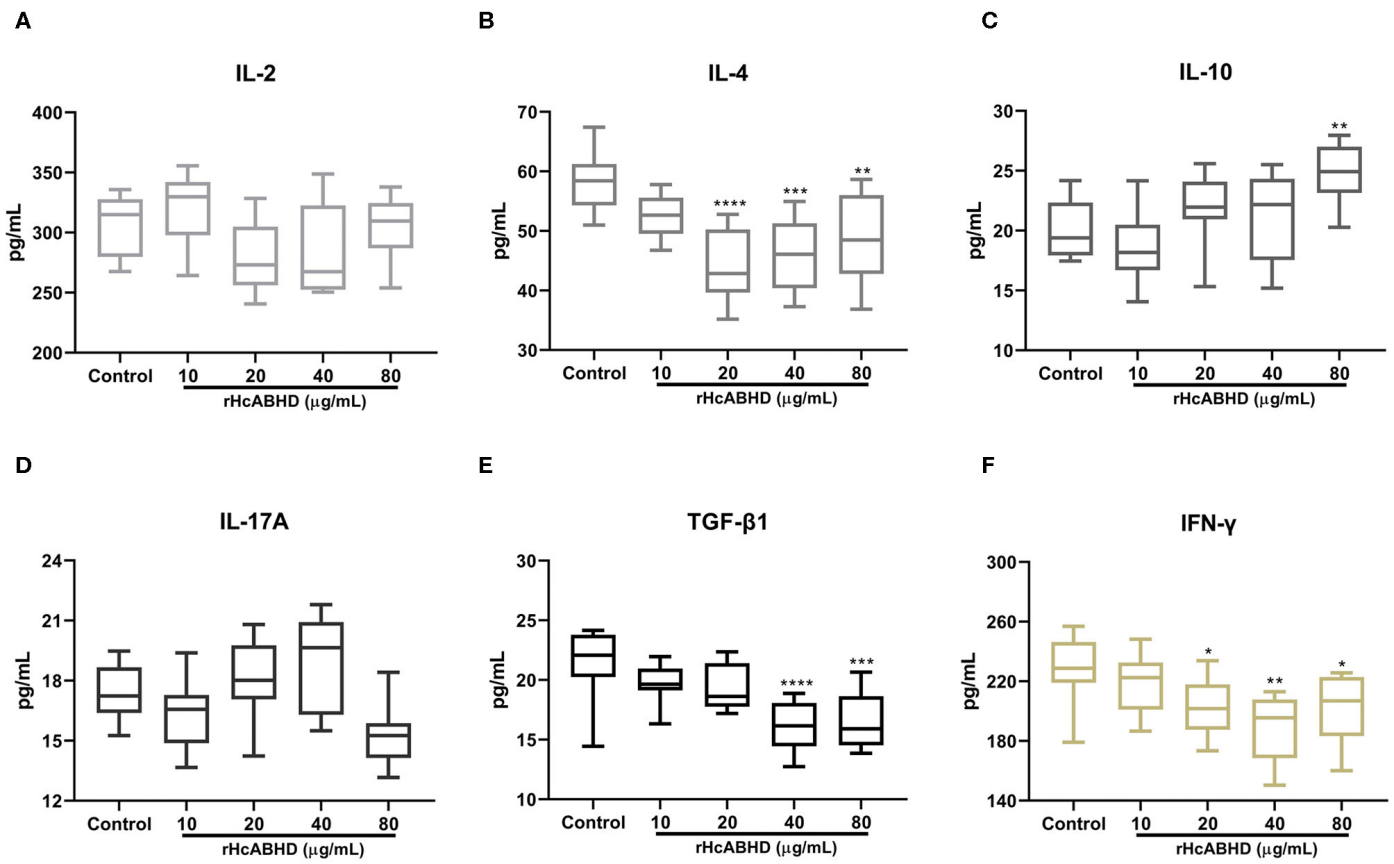
The rHcABHD stimuli altered T cells cytokine secretion profiles *in vitro*. Goat T cells were incubated in the presence or absence of rHcABHD proteins for 24 h. The secretions of IL-2 **(A)**, IL-4 **(B)**, IL-10 **(C)**, IL-17A **(D)**, IFN-γ **(E)**, and TGF-β1 **(F)** in the culture supernatant were determined by ELISA. Data are presented as Minimum to Maximum, *P < 0.05, **P < 0.01, ***P < 0.001, ****P < 0.0001 vs. control group. Each experiment was run in triplicate.

To investigate the modulatory effects of rHcABHD on T cell cytokine productions, IL-2, IL-4, IL-10, IL-17A, IFN-γ, and TGF-β1 secretions were determined by ELISA. The results showed that goat T cells exposed to rHcABHD stimuli altered their cytokine production profiles. The productions of IL-4 and IFN-γ were predominantly inhibited by the stimulation with 20 μg/mL (*P* < 0.0001 and *P* < 0.05, respectively), 40 μg/mL (*P* < 0.001 and *P* < 0.01, respectively) and 80 μg/mL (*P* < 0.01 and *P* < 0.05, respectively) of rHcABHD proteins (Figures 4B,F). Meanwhile, IL-10 secretion was significantly promoted by the treatment with 80 μg/mL of rHcABHD (*P* < 0.01) (Figure 4C), whilst 40 μg/mL (*P* < 0.0001) and 80 μg/mL (*P* < 0.001) of rHcABHD inhibited TGF-β1 production (Figure 4E). Intriguingly, all the tested doses of rHcABHD (10, 20, 40, and 80 μg/mL) had no significant effects on the secretions of IL-2 or IL-17A as compared with the control group (Figures 4A,D).

## Discussion

Parasitic helminths have evolved sophisticated and highly integrated mechanisms to encourage the coexistence with hosts (35). They could release ES proteins into the host environment to suppress or subvert host immune response so as to ensure their survival (36). As an individual ES component derived from HcESPs which interacted with host T cells, the HcABHD gene was completely cloned, and the rHcABHD protein was successfully obtained in the present study. Although ABH superfamily members display surprisingly low sequence identity among all three domains of life, ABHD proteins are relatively conservative with each other within the ABHD family (37). Based on the phylogenetic analysis with vertebrate orthologs and parasite homologs, HcABHD protein was supposed to belong to ABHD17 subfamily, namely ABHD17A, ABHD17B, and ABHD17C. Mammalian ABHD17 proteins are novel depalmitoylating enzymes with substrate specificity to N-Ras and postsynaptic density protein 95 (PSD95). The catalytic activities of ABHD17 proteins are indispensable for N-Ras depalmitoylation and subcellular re-localization (38), whereas ABHD17 proteins could also accelerate PSD-95 palmitate turnover and control synaptic PSD-95 amount in neurons (39). Currently, to our knowledge, very little is known about the regulation, biochemical and physiological functions of parasite ABHD17 proteins. Given that rHcABHD was recognized by sera from infected goats and native HcABHD protein was appraised by specific rat anti-HcABHD IgG, HcABHD protein with outstanding immunogenicity could be a valuable candidate for the development of diagnostic assays in *H. contortus* infection. Additionally, native HcABHD protein was localized in the platymyrian muscle cells under the cuticle and gut region of adult worms, indicating that HcABHD protein could be excreted or secreted through the worm cuticle or gut actively/passively into the host. Combined with the amounting mRNA expressions of HcABHD in *H. contortus* life-cycle, HcABHD may play a crucial role in the development, invasion, and survival of *H. contortus*.

Like other ABH superfamily members, HcABHD protein with a nucleophile-His-acid catalytic triad may have the potential to catalyze the hydrolysis of certain substrates with different chemical composition or physicochemical properties (40). In this study, we determined that rHcABHD protein exerted strong capability of generating glycerol from triglycerides *via* catalyzing the cleavage of the ester bonds as a lipid metabolizing enzyme. In addition, we also validated the positive *in vitro* interaction of rHcABHD with goat T cells by immunocytochemistry assays based on our preliminary LC-MS/MS analysis. Although we employed proteomic approaches to gain a variety of interacting proteins such as kinases, hydrolases, proteases, lipases and phosphatases, the exact molecule(s) in HcESPs which were involved in the regulation of T cell immune response still need to be further investigated. Given the catalytic biological activities in lipid metabolism and the interaction with host T cells, HcABHD could be an essential immunomodulator acting at the parasite-host interface. Intriguingly, the treatments with rHcABHD dramatically inhibited T cell viability and induced cell apoptosis. Caspases are a family of cysteine proteases and the central regulators of apoptosis which is characterized by nuclear condensation, cell shrinkage, membrane blebbing, and DNA fragmentation (41). Initiator caspases like caspase-8, and−9 coupled to pro-apoptotic signals get activated and cleaved, and lead to the activation of downstream effector caspases such as caspase-3, which in turn execute apoptosis by cleaving cellular proteins. Activation of death receptor result in the activation of caspase-8 in intrinsic apoptosis pathway, whereas cytochrome c released from damaged mitochondria initiate the activation of caspase-9 in extrinsic apoptosis pathway (42). In current study, rHcABHD stimuli notably upregulated the transcription of caspases-8,−9, and−3, indicating a potential mechanism underlying HcABHD-induced intrinsic and extrinsic apoptosis of T cells.

Cell apoptosis, proliferation, and cell cycle are fundamental and ultimately linked cellular events. Therefore, we further testified the immunomodulatory potential of rHcABHD on T cell proliferation and demonstrated that rHcABHD protein strongly suppressed T cell growth in a dose-dependent manner. Eukaryotic cell growth and division are controlled by G1/S checkpoint and G2/M checkpoint to ensure proper timing of cell cycle phase to the next, which involves a coordinated set of proteins monitoring uncontrolled cell division and propagation of damaged DNA (43). The G1/S checkpoint controlled by two kinase complexes CDK4/6-Cyclin D and CDK2-Cyclin E initiates the passage of the G1 phase into the DNA synthesis S phase (44), while the G2/M checkpoint serves to restrain DNA-damaged cells into mitosis (M-phase) *via* the Cyclin B-CDK1 kinase complex (45). In this study, flow cytometry analysis revealed that rHcABHD caused cell cycle arrested at G1 phase, but not G2/M phase. Meanwhile, the transition through G1 phase into S phase in T cells was prevented by rHcABHD stimuli *via* the downregulation of CCND1, CDK4/6, and CCNE1 transcriptions. Dynamic cycle of de-palmitoylation and palmitoylation are reversible modification processes of proteins involving a plethora of biological progresses including protein trafficking, subcellular localization, and cell signaling (46). Ras proteins are small GTPases engaged in signal transduction of growth factors to control cell differentiation and growth. Palmitoylated Ras is distributed toward the plasma membrane after post-translational modifications, whereas depalmitoylated Ras tends to accumulate in the Golgi where it undergoes palmitoylation and recycle to plasma membrane again (47). Therefore, disruption of Ras palmitoylation/depalmitoylation cycle that suppressed cell growth and development is targeted for cancer therapy (48). As mammal ABHD17 homologs and exogenous depalmitoylase, the external stimuli of rHcABHD may disrupt the balance of Ras palmitoylation cycle, block Ras membrane association, and interfere RAS signaling along with its downstream effectors in host T cells, particularly the kinase cascades PI3K-AKT-MTOR and RAF-MEK-ERK (49). Collectively, this could be one of the mechanisms of how HcABHD targets the regulation of host T cell cycle, apoptosis, and proliferation.

Immunomodulatory activities generated by ES proteins of parasitic helminth exhibit several predominant features: blocking pro-inflammatory and Th1 cytokines including IFN-γ and IL-2, inducing anti-inflammatory cytokines like IL-10 and TGF-β, regulating Th2 responses such as the secretion of IL-4, and modulating Treg and Th17 responses (36, 50). Consistent with these findings, rHcABHD stimuli significantly suppressed the secretions of IL-4 and IFN-γ, but not IL-2, indicating the critical controls of HcABHD on Th2 and pro-inflammatory responses. Several ES immunomodulators, such as *Toxascaris leonina* galectin-9 homolog (51), *Trichinella spiralis* 7C2C5 antigen and 53-kDa protein (52), restrained inflammation reactions by inhibiting Th1 and Th2 cytokine production *via* enhanced TGF-β and IL-10 generation. However, in this study, high doses of rHcABHD upregulated IL-10 production, whereas downregulated TGF-β1 secretions. TGF-β signaling consisting of TGF-β/activin and BMP pathways plays a critical role in the regulation of cell growth, differentiation, and development, and these two pathways are modulated by PI3K/AKT/MAPK signaling at a number of levels (53). Thus, external rHcABHD stimuli-induced downregulation of TGF-β1 secretion may exacerbate T cell apoptosis involving PI3K/AKT/MAPK signaling, which might in turn affect TGF-β signaling and inhibit T cell growth. Intriguingly, we hereby observed the modulatory effects of rHcABHD protein on cell growth and cytokine production profiles. However, whether the alteration of IL-4, IL-10, TGF-β1, and IFN-γ secretions was resulted from rHcABHD stimuli-caused less alive T cells, or more hyporesponsive T cells, or different T cell subsets, remains unclear and more mechanistic studies are still needed.

ABHD17 proteins (A, B, and C) exhibit a dizzying array of function properties. They are termed as thioesterases or depalmitoylases that engage in the enzymatic process of protein depalmitoylation, like acyl protein thioesterase 1 and 2 (46), and also categorized as lipases or serine hydrolase with the characterization of the specific α/β-hydrolase domain (54). Therefore, ABHD17 family members could be defined as functionally malleable proteins with diverse biological activities attributed to their unexpected catalytic efficiency. In this study, we identified HcABHD as a lipase and immunomodulator acting at the parasite-host interface. However, with limited availability of goat immune reagents, we hereby only validated the transcription levels of several key factors involved in cell apoptosis and growth. More detailed mechanistic networks at the protein level, alongside associated pathways, merit further investigation. Clearly, future efforts are imperative to explore more detailed substrate profiles of HcABHD, the exact molecule(s) that regulated by or interacted with HcABHD, and its prophylactic potential for anti-*H. contortus* vaccine development.

## Data Availability Statement

All datasets generated for this study are included in the article/Supplementary Material.

## Ethics Statement

The animal study was reviewed and approved by Science and Technology Agency of Jiangsu Province (Approval No. SYXK (SU) 2010–0005). All animal experiments were performed in strict compliance with the Guidelines of the Animal Welfare Council of China. All efforts were made to minimize the suffering of animals, and daily health checks were performed throughout the experiments.

## Author Contributions

ML performed the laboratory tests, analyzed the data and drafted the manuscript. XL directed, coordinated, and managed the project, and edited the manuscript. XT and A-LT contributed to the *in vitro* studies and assisted with cell cultures. CL helped in the implementation of the study and edited the manuscript. RY, XS, and LX provided new analytical reagents and tools. All authors read and approved the final manuscript. All authors contributed to the article and approved the submitted version.

## Conflict of Interest

The authors declare that the research was conducted in the absence of any commercial or financial relationships that could be construed as a potential conflict of interest.

## Supplementary Material

Supplementary Figure 1 Goat T cells sorting by MACS. The purity of isolated T cells was determined by flow cytometry analysis, and was above 95% as indicated before **(A)** and after **(B)** MACS sorting.

Supplementary Table 1 Primer sequences for HcABHD transcription analysis.

Supplementary Table 2 Primer sequences for the transcription analysis of apoptosis and cell cycle.

## Abbreviations

ABH: α/β-hydrolase
ABHD: α/β-hydrolase domain
ES: excretory and secretory products
HcESPs: *Haemonchus contortus* excretory and secretory proteins
HcABHD: *H. contortus* α/β-hydrolase domain protein
IL: interleukin
IFN: interferon
TGF: Transforming growth factor
LC-MS/MS: liquid chromatography-tandem mass spectrometry
PBMCs: peripheral blood mononuclear cells
CDK: cyclin-dependent kinase
Akt: AKT8 virus oncogene cellular homolog
MAPK: mitogen-activated protein kinase
Erk: extracellular signal-regulated kinase.
