# Supplementary figures and images for: A Novel α/β Hydrolase Domain Protein Derived From Haemonchus contortus Acts at the Parasite-Host Interface
Source: Front Immunol. 2020 Jun 30;11:1388. doi: 10.3389/fimmu.2020.01388 (PMC7338770; doi:10.3389/fimmu.2020.01388)

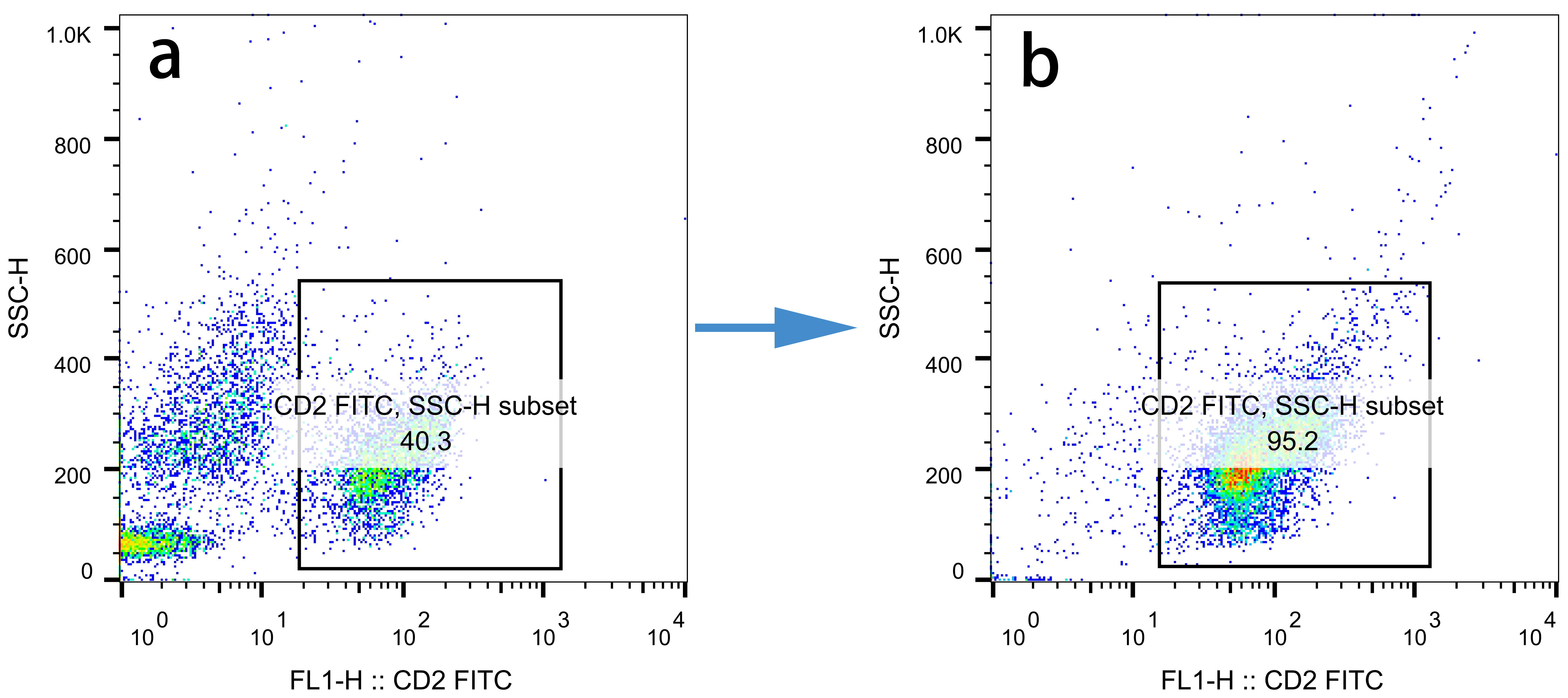

Supplement: Supplementary Figure 1 — Goat T cells sorting by MACS. The purity of isolated T cells was determined by flow cytometry analysis, and was above 95% as indicated before (A) and after (B) MACS sorting. [file Image_1.TIF]
