# Supplementary material for: A Novel α/β Hydrolase Domain Protein Derived From Haemonchus contortus Acts at the Parasite-Host Interface
Source: Front Immunol. 2020 Jun 30;11:1388. doi: 10.3389/fimmu.2020.01388 (PMC7338770; doi:10.3389/fimmu.2020.01388)
Supplement: Supplementary Table 1 — Primer sequences for HcABHD transcription analysis. [file Table_1.DOCX]

**Supplementary Table 1: Primer sequences for HcABHD transcription analysis**

| **Gene Name** | **Primer Sequence (5’-3’)** | **Reference** | **Size (bp)** |
| --- | --- | --- | --- |
| β-Tubulin | F: TGCTATGTTCCGTGGTCGTATG  R: CGGCAGTCTTAACGTTGTTTGG | [27] | 116 |
| HcABHD | F: GGAAGACCATCGGAGAAGAATC  R: CGTACCTATGGATTGCCCATAA | EMBL: HF964273.1 | 117 |
