# Supplementary material for: A Novel α/β Hydrolase Domain Protein Derived From Haemonchus contortus Acts at the Parasite-Host Interface
Source: Front Immunol. 2020 Jun 30;11:1388. doi: 10.3389/fimmu.2020.01388 (PMC7338770; doi:10.3389/fimmu.2020.01388)
Supplement: Supplementary Table 2 — Primer sequences for the transcription analysis of apoptosis and cell cycle. [file Table_2.DOCX]

**Supplementary Table 2: Primer sequences for the transcription analysis of apoptosis and cell cycle**.

| **Gene Name** | **Primer Sequence (5’-3’)** | **Reference** | **Size (bp)** |
| --- | --- | --- | --- |
| beta-actin | F: CACCACACCTTCTACAAC  R: TCTGGGTCATCTTCTCAC | [29] | 106 |
| Caspase 3 | F: CATTATTCAGGCCTGCCGAG  R: CTCGAGCTTGTGAGCGTACT | [30] | 220 |
| Caspase 8 | F: TTAGCATAGCACGGGAGCAG  R: GTCAGCTCATAGATGGGGGC | [30] | 280 |
| Caspase 9 | F: GGGAAATGCTGATCTGGCCT  R: CAGCCGTGAGAGAGGATGAC | [30] | 279 |
| CCND1 | F: GGTCCTGGTGAACAAACTC  R: TTGCGGATGATCTGCTT | [31] | 114 |
| CDK4 | F: CGTTGGCTGTATCTTTGC  R: GATTCGCTTGTGTGGGTT | [31] | 256 |
| CDK6 | F: AGAGTGATTGCAGCTTTATGTCCA  R: TGCCCAGGTTGCTCACTTC | [31] | 157 |
| CCNE1 | F: GGGACAAGCACCTTATGCAAC  R: GTGTTGCCATATACCGATCAAAGA | [31] | 153 |
| CDK2 | F: CTGCACCGAGACCTTAAACCTCA  R: GCTCGGTACCACAGAGTCACCA | [31] | 140 |
| Akt1 | F: GTACTCCTTCCAGACACACGACC  R: TACACCACGTTCTTCTCCGAG | [32] | 174 |
| p38 | F: GAAAGCAGGGACCTCCTTATAG  R: CAGACACTCAAGACTCCATCTC | [33] | 109 |
